# The GABAergic system in prefrontal cortex and hippocampus modulates context-related extinction learning and renewal in humans

**DOI:** 10.1007/s11682-016-9662-y

**Published:** 2016-12-07

**Authors:** Silke Lissek, Anne Golisch, Benjamin Glaubitz, Martin Tegenthoff

**Affiliations:** Department of Neurology, BG University Hospital Bergmannsheil, Ruhr-University Bochum, Bochum, Germany

**Keywords:** Extinction, Renewal, GABA, Hippocampus, Prefrontal cortex

## Abstract

Context-related extinction learning and renewal in humans is mediated by hippocampal and prefrontal regions. Renewal is defined as the reoccurrence of an extinguished response if the contexts present during extinction learning and recall differ. Animal studies implicate hippocampal γ-aminobutyric acid (GABA) A receptors in extinction and renewal. However, human studies on GABAergic mechanisms in extinction learning are lacking. In this fMRI study, we therefore investigated the role of the GABAergic system in context-related extinction learning and renewal. Participants treated with the GABA A agonist lorazepam prior to extinction learning were impaired in encoding changed associations during extinction learning, regardless of context, and in retrieving extinction associations during recall. In contrast, retrieval of associations learned during acquisition was largely unaffected, which led to reduced genuine renewal, since acquisition associations were retrieved context-independently. These deficits, which were presumably due to weak encoding of extinction associations, were related to altered BOLD activation in regions relevant for context processing and retrieval, as well as response selection: reduced activation in bilateral PFC and hippocampus during extinction learning and recall, and increased ventromedial/orbitofrontal cortex activation during recall. Our findings indicate that the GABergic system is involved in context-related extinction learning and recall in humans, by modulating hippocampus-based context processing and PFC-based processing of changed associations and subsequent response selection.

## Introduction

Human and animal research on extinction learning has identified amygdala, insula, prefrontal cortex and hippocampus as important regions participating in both fear extinction (Herry et al. 2010; Sehlmeyer et al. 2009), and non-fear related extinction (Todd et al. 2014). Hippocampus and ventromedial / orbitofrontal cortex in particular have prominent roles in processing context-related extinction learning and retrieval (Good and Honey 1991; Kalisch et al. 2006; Lissek et al. 2013; Maren and Holt 2000; Milad et al. 2007; Quirk et al. 2000; Ridder et al. 2009; Vianna et al. 2004). The level of context processing in an individual can be inferred from their retrieval performance after extinction learning in a context different from that present during recall. In many cases, such a paradigm induces renewal, which is defined as the reoccurrence of a previously extinguished response if the contexts of extinction learning and recall differ (Bouton and Ricker 1994). The prerequisites for renewal to occur, e.g. context encoding during extinction learning and context retrieval in subsequent recall, were found mediated by hippocampus and vmPFC, respectively, suggesting that these regions have a crucial role in evoking renewal (Lissek et al. 2013). Already during initial conditioning or acquisition, hippocampal activation is more prominent in participants who later show renewal (Lissek et al. 2016), a finding that underlines the important role of hippocampus for context consideration throughout conditioning and extinction.

However, the distinct contributions of neurotransmitters in these extinction-relevant brain regions to human extinction learning and particularly to renewal are relatively poorly understood. Studies in humans showed that stimulation of the noradrenergic (NA) system enhanced, and blockade of the dopaminergic (DA) system impaired, extinction learning proper (Lissek et al. 2015a; Lissek et al. 2015b), while hippocampus and vmPFC/OFC showed opposing activation patterns: increased activity during stimulation of the noradrenergic system and reduced activity during dopaminergic blockade. Yet, neither intervention had a significant impact upon the level of renewal, despite moderate increases or decreases with NA agonism and DA antagonism, respectively, that matched the effects of the pharmacological manipulation upon hippocampal activation.

A potentially important candidate for modulating extinction learning and renewal is the inhibitory neurotransmitter γ-aminobutyric acid (GABA) and its action at GABA A receptors (Singewald et al. 2015). Animal studies revealed differential effects of GABAergic manipulations upon fear extinction learning and renewal depending on the brain region investigated: Local infusion of the GABA A agonist muscimol into rat hippocampus prior to extinction learning and recall disrupted extinction learning and renewal, respectively (Corcoran and Maren 2001; Corcoran et al. 2005; Hobin et al. 2006). Renewal of appetitive responding, however, was found unaffected after local muscimol infusions prior to recall (Campese and Delamater 2014). In contrast, GABA A agonists in prefrontal cortex appear to have either facilitating effects or no effects at all upon fear extinction learning: infusion of muscimol into rat infralimbic PFC enhanced (Akirav et al. 2006) or did not affect (Chang and Maren 2011; Laurent and Westbrook 2008) extinction learning. However, local infusion of the GABA A antagonist picrotoxin prior to contextual fear extinction was found to accelerate extinction (Thompson et al. 2010). Also in appetitive instrumental extinction, local GABA A agonism in rat prelimbic and infralimbic cortex, respectively, was found to have no impact upon extinction and recall (Mendoza et al. 2015), comparable to appetitive Pavlovian extinction, where infusion of muscimol into OFC prior to extinction did not impair extinction behavior (Panayi and Killcross 2014). One study using systemic muscimol in rats reported a disruption of extinction and consolidation for taste aversion learning (Disorbo et al. 2009).

In summary, manipulation of the rat GABAergic system did not affect appetitive conditioning, regardless of target area. In contrast, adequate GABAergic processing in hippocampus appears to be required for context-related extinction and renewal, as administration both before extinction learning and recall demonstrated, indicating an important role for hippocampal GABA in context processing. Findings from GABAergic modulation of rat prefrontal regions overall suggest that they have no crucial role in altering existing associations.

However, human studies evaluating contributions of the GABAergic system to extinction learning and renewal are lacking. The few experiments that modulated the GABAergic system in humans demonstrated that GABA A agonism using benzodiazepines impaired visual paired associate learning (Pietrzak et al. 2012) as well as memory for spatial contextual information (Mintzer and Griffiths 1999) and enhanced reconsolidation of associative learning (Rodríguez et al. 2013) - all these are aspects of learning that are potentially relevant for context-related extinction.

In the present study, we therefore investigated the effects of GABA A agonism upon learning performance and brain activation patterns in context-related extinction learning and the renewal effect. In an associative learning task featuring an ABA design that reliably evokes a renewal effect, healthy human participants learned associations between cues presented in particular contexts, and outcomes. Prior to extinction learning of these associations, participants were treated with the GABA A agonist lorazepam. Based on animal research findings on hippocampal and prefrontal GABA A agonism, as well as on results from the few human studies using systemic GABA A agonism, we hypothesized that systemic GABA A agonism in humans would interfere with adapting previously established cue-outcome associations, increasing error rates during extinction learning. Concerning hippocampal context processing, we expected the GABA A agonist to impair context encoding during extinction learning, which would reflect in a reduction of the renewal effect during recall: due to a lack of context encoding we expected responses to preferrably contain associations learned during extinction, for both novel and familiar contexts. In parallel, we expected the GABA A agonist to modify activation in extinction-relevant regions, with particularly prominent effects upon hippocampus and prefrontal regions.

## Materials and methods

### Participants

54 healthy right-handed volunteers (29 women, 25 men), mean age 25.54 years +/− 3.98 years st.dev., range 19–42, without a history of neurological disorders, participated in this study after giving written informed consent. Prior to the experiments, participants received handouts informing them about the fMRI procedures and the GABA agonist. The protocol was approved by the Ethics Committee of the Ruhr-University Bochum. The study conforms to the Code of Ethics of the World Medical Association (Declaration of Helsinki). The participants received a monetary compensation for their participation (in the amount of € 60). Participants were randomly assigned to the experimental (GABA A agonist) and control (Placebo) groups.

### Predictive learning task

The predictive learning task that we used in this study was adapted for use in an fMRI setting from a task originally devised by Üngör & Lachnit (Üngör and Lachnit 2006), which constitutes an established paradigm to study associative extinction learning and the renewal effect without a fear component. By means of the task design, a renewal effect can be reliably evoked, as demonstrated in a number of behavioral studies (Lachnit et al. 2008; Lucke et al. 2013; Nelson and Callejas-Aguilera 2007; Üngör and Lachnit 2006; Rosas and Callejas-Aguilera 2006). Further studies have investigated other phenomena of context-related extinction learning using this paradigm of the predictive learning task - e.g. extinction in multiple contexts (Bustamante et al. 2016; Glautier et al. 2013) or differently composed contexts (Lucke et al. 2014), as well as partial reinforcement and context switch effects (Abad et al. 2009; Rosas and Callejas-Aguilera 2006). In imaging and pharmacological studies, the predictive learning task has been used to investigate areas active during extinction and renewal, the role of stress and of the dopaminergic and noradrenergic systems for extinction and renewal (Hamacher-Dang et al. 2013; Kinner et al. 2016; S. Lissek et al. 2015a; Lissek et al. 2015b; Lissek et al. 2013, 2016). The task is designed to allow for learning of associations between cues and outcomes with or without encoding of the context, since regarding or ignoring the context does not impact the ability to learn the task. In the predictive learning task, we use two conditions: a) the experimental ABA condition, in which extinction is performed in a context different from that present during acquisition and recall, and b) the control AAA condition, in which all learning occurs in an identical context. While the ABA condition serves to evoke a renewal effect during recall, the AAA condition serves to control extinction learning success - a high number of errors in AAA recall signals impaired extinction learning, which may relativize whether the responses observed in ABA actually indicate genuine renewal.

In the predictive learning task, participants were asked to put themselves in the position of a physician and predict whether various items of food (vegetables or fruits) served in different restaurants would lead to the aversive consequence of stomach trouble in their patient. By describing this situation, the written instructions provided an implicit differentiation between the cue and the context. To make sure all participants understood the task, participants were prompted to repeat the content of the instructions after reading, and ask further questions regarding aspects they did not understand. In general, data sets from participants not following the instructions ( e.g. using wrong response buttons), or delivering an acquisition performance that shows they do not understand the task, are excluded from the analysis. In the present study, no data sets had to be excluded.

**Fig. 1:**
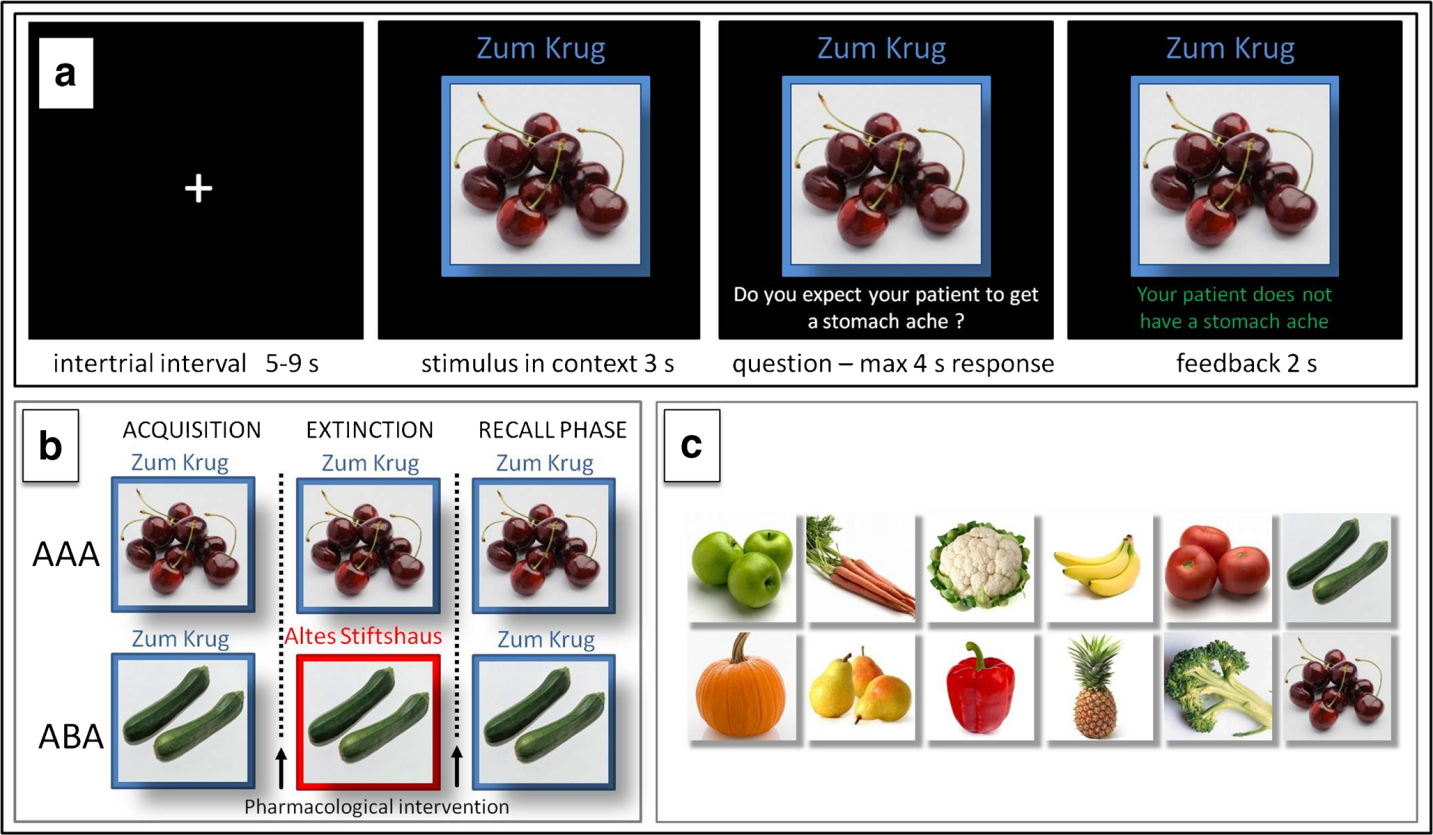
Predictive Learning Task. **a** example of a trial: Participants learned to predict whether certain kinds of food, eaten in a certain restaurant, would cause a stomach ache or not. After an intertrial interval of 5–9 s the stimulus was presented in its context for 3 s, then a question was superimposed on the screen “Will the patient get a stomach ache?” for maximum 4 s response time. Feedback was shown for 2 s, providing the correct answer, e.g. “The patient does not have a stomach ache.” **b** design of the predictive learning task. In condition AAA, extinction occurs in the same context as acquisition. In condition ABA, extinction occurs in a context different from that during acquisition. In both conditions, the final test for the renewal effect is performed in the context of acquisition during the recall phase. **c** 12 food images used as stimuli

The learning process consisted of the successive phases of a) acquisition of associations, b) extinction and c) recall phase (see Fig. 1). During the acquisition phase, participants learned to associate items of food with specific consequences. In each trial, one of twelve stimuli was presented to the participant in one of two different contexts (indicated by the restaurant names “Zum Krug” and “Altes Stiftshaus” and a frame in either red or blue color). The stimulus in its context was presented for 3 s on its own, then a question asking whether the patient will develop a stomach-ache was superimposed, with the response options ‘Yes’ or ‘No’. Response time was 4 s, participants responded by pressing the respective button on an fMRI-ready keyboard (Lumitouch, Photon Control Inc. Canada). After the response, or in case of a missing response after expiration of the response time, a feedback with the correct answer was displayed for 2 s, i.e. “The patient has a stomach-ache” (in red font) or “The patient does not have a stomach-ache” (in green font). The actual response of the participant was not commented upon. Six stimuli were presented per context. Each stimulus was presented in eight trials, resulting in an acquisition phase consisting of 96 trials.

During the extinction phase (96 trials), half of the stimuli were presented in the same context as during acquisition (condition AAA - no context change - 48 trials) and the other half in the other context (condition ABA - context change, designed to induce a renewal effect - 48 trials) in randomized order. For actual ‘extinction stimuli’, the consequence of stomach trouble changed and the new consequence had to be learned, for ‘distractor stimuli’, which were introduced in order to make overall learning more difficult, the consequence during extinction remained unchanged. In all other respects, trials were identical to those during acquisition.

During the recall phase (60 trials), all stimuli were presented once again in the context of acquisition (5 presentations per stimulus). With the exception that during the recall phase no feedback with the correct response was given, trials were identical to those during acquisition. The omission of a feedback serves to prevent further learning during the recall phase, and thus prevents carry-over effects. Therefore, the relative high number of stimulus presentations, required for imaging, does not affect the results.

In the predictive learning task, the context consists of a restaurant name and a colored frame around the cue (food stimulus). There is a debate about whether stimuli have certain properties that allow for their apriori classification as cues or as contexts (Nadel and Willner 1980). Cues are supposed to be discrete and rapidly changing, whether context is thought to be diffuse and slowly changing. An alternative view is that there is not categorical difference between discrete cues and contextual information, and that context is learned (Myers and Gluck 1994), so that the differentiation between them comes about through their associations with the US: cues, but not contexts have high relative contingency and contiguity with the US. Our contexts match the above criteria as they were slowly changing and comparably diffuse: they appeared with considerably higher frequency than individual cues, because they were presented again and again with varying cues, and thus came to be associated with the consequences of both stomach ache and no stomach ache in a randomized manner. They were not directly associated with the US and therefore could not predict the US.

### Procedure

The experiment was performed on two successive days in order to avoid effects of the GABA agonist upon consolidation of the acquisition phase of the predictive learning task.

On the first day, participants performed the acquisition phase of the predictive learning task in a first fMRI session. On the second day, the GABA A agonist lorazepam (TAVOR ®, Pfizer) was administered orally in a single dose of 2.5 mg. Control participants received an identical-looking placebo. After drug administration, participants rested for 90 min. The second fMRI session consisting of the extinction and the recall phase was performed in a time window of about 90 to 150 min after administration of the drug. The task timing was based on the phase of peak plasma levels for lorazepam (Greenblatt et al. 1976).

### Imaging data acquisition

Functional and structural brain scans were acquired using a whole-body 3 T scanner (Philips Achieva 3.0 T X-Series, Philips, The Netherlands) with a 32-channel SENSE head coil. Blood-oxygen level dependent (BOLD) contrast images were obtained with a dynamic T2* weighted gradient echo EPI sequence using SENSE (TR 3200 ms, TE 35 ms, flip angle 90°, field of view 224 mm, slice thickness 3.0 mm, voxel size 2.0 × 2.0 × 3.0 mm). We acquired 45 transaxial slices parallel to the anterior commissure — posterior commissure (AC-PC) line which covered the whole brain. High resolution structural brain scans of each participant were acquired using an isotropic T1 TFE sequence (field of view 240 mm, slice thickness 1.0 mm, voxel size 1x1x1 mm) with 220 transversally oriented slices covering the whole brain. The task was presented to the participants via fMRI-ready LCD-goggles (Visuastim Digital, Resonance Technology Inc., Northridge, CA, USA) connected to a laptop which ran specific software programmed in Matlab. Responses were given by means of an fMRI-ready keyboard (Lumitouch response pad, Photon Control Inc., Canada).

### Imaging data analysis

For preprocessing and statistical analysis of fMRI data we used the software Statistical Parametric Mapping (SPM), Version 8 (Wellcome Department of Cognitive Neurology, London, UK), implemented in Matlab R2008a (Mathworks, Natick, MA, USA). Three dummy scans, during which the BOLD signal reached steady state, preceded the actual data acquisition of each session, thus preprocessing started with the first acquired volume. Preprocessing on single subject level consisted of the following steps: slice timing correction to account for time differences due to multislice image acquisition; realignment of all volumes to the first volume for motion correction; spatial normalization into standard stereotactic coordinates with 2x2x2 mm^3^ using an EPI template of the Montreal Neurological Institute (MNI) provided by SPM, smoothing with a 6 mm full-width half-maximum (FWHM) kernel, in accordance with the standard SPM procedure. The acceptable limit for head motion was 2 mm for translational movements and 0.5° for rotational movements.

In a first level single subject analysis we calculated activation during extinction and recall phases, contrasted against baseline. We used an anatomically defined mask which was constructed using the software MARINA (BION Bender Institute of Neuroimaging, University of Giessen, Germany) (Walter et al. 2003) based on the AAL (Automated Anatomical Labeling) atlas (Tzourio-Mazoyer et al. 2002). The mask contained, as a priori regions of interest, prefrontal cortex (superior, middle and inferior frontal gyrus), hippocampus, amygdala and insula. All data contained in this combined mask were analyzed together in a single analysis.

We used an event-related design, modeling the events of each trial (onsets of image, questions, feedback) using distinct stick functions convolved with the canonical hemodynamic response function in SPM. Contrasts used for the second-level analyses were based on the onset of the image of the context-cue compound at the beginning of a trial, compared to baseline. For the contrasts of the extinction learning phase shown in the results, only those stimuli for which the stomach trouble prediction, learned during acquisition, changed during the extinction phase were considered in the analysis (i.e. extinction stimuli or consequence change stimuli).

The contrast images from the single subject analyses were entered into second-level random-effects analyses to compare BOLD activation in the experimental and control groups for extinction learning and recall phases in the ABA and AAA conditions, using an FWE-corrected threshold of *p* < 0.05 cluster-level. In particular, we performed between-group analyses (two-sample t-tests for independent samples) contrasting the activation patterns of the GABA and PLAC groups separately in the phases of ABA and AAA extinction learning as well as ABA and AAA recall (contrasts PLAC > GABA and GABA > PLAC). These analyses served to show in which extinction-relevant regions the GABA A agonist evoked altered activation.

### Behavioral data analysis

For all three learning phases, log files were written that contained information on response latency, response type and correctness of response, from which we calculated overall error rates during acquisition and extinction learning, moreover specific error rates for stimuli with consequence change (CC) and distractor stimuli during extinction learning. For calculation of the renewal effect, during the recall phase only responses to stimuli with consequence change (extinction stimuli) were analyzed. The behavioral renewal effect in the predictive learning task is supposed to occur only in the condition ABA, due to the context change introduced during extinction learning. In case of renewal, associations learned during acquisition in context A will reappear in the recall phase which is again performed in context A, while extinction was performed in context B.

In contrast, the AAA condition constitutes a control condition for extinction learning, since here all learning phases are performed in an identical context. If extinction learning is successful, responses during the recall phase will reflect the associations learned during extinction. Only if extinction learning is impaired, responses in the AAA recall phase will reflect associations learned during acquisition.

Errors in acquisition and extinction learning were defined as responses stating the incorrect association between the context-cue-compound and the consequence. During the recall phase, a response that referred to the association which was correct during acquisition constituted an error in the AAA condition and a renewal response in the ABA condition. Statistical analyses were performed using the IBM SPSS Statistics for Windows software package, version 22.0 (Armonk, NY: IBM Corp.). All results are quoted as mean +/− s.e.m., unless stated otherwise.

For the behavioral analyses in which we compared participants who showed or did not show renewal, participants of the GABA and PLAC groups were assigned to their respective “renewal” subgroup if they showed at least 10% renewal responses during recall.

For basic behavioral analyses, we calculated between-groups t-tests for independent samples comparing GABA and PLAC groups regarding performance in acquisition, extinction learning and recall, moreover for response times. In order to analyze the learning curve in extinction learning, we calculated an ANOVA for the complete group of participants with the between-subject factor treatment and the within-subject repeated-measures factor learning block.

## Results

### Behavioral results

#### Learning performance during acquisition

**Fig. 2:**
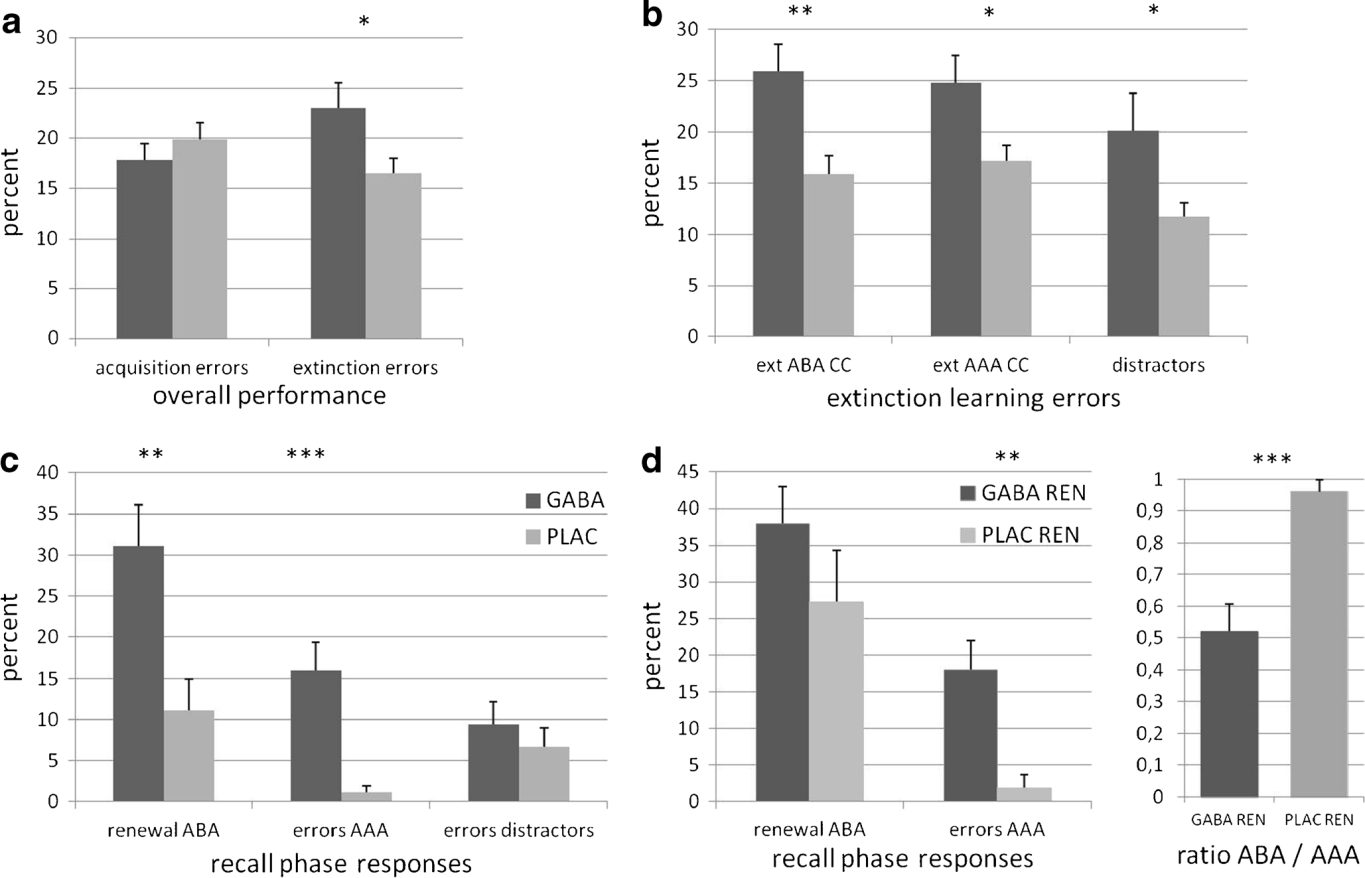
Learning performance in the predictive learning task. **a** Overall performance in acquisition and extinction of the GABA (*black*) and PLAC (*gray*) groups: while the groups did not differ in initial learning prior to drug administration, the GABA group made significantly more errors during extinction learning. **b** Extinction learning performance for the ABA and AAA extinction trials (CC = consequence change, i.e. those trials in which the consequence of stomach trouble changed during the extinction phase) and for the distractor trials: GABA participants made more errors in learning the changed associations both in the familiar and the novel context. Moreover, they made more errors in distractor trials requiring retrieval of the associations learned during acquisition. **c** Performance in the recall phase: GABA participants responded more frequently with associations correct during acquisition than PLAC. In retrieval of distractor trials, the groups did not differ. **d** Performance in the recall phase, only participants who showed renewal: GABA and PLAC participants exhibited a similar level of renewal responses, while GABA participants made significantly more AAA errors, responding with associations correct during acquisition. Accordingly, the ratio of acquisition responses in ABA and AAA recall trials differs significantly between the groups, indicating that the PLAC group’s behavior in recall reflected genuine renewal that considered the context, while the GABA group’s behavior did not

During the first acquisition of the predictive learning task, which was performed prior to drug administration, GABA and PLAC groups did not differ in their learning performance (*t*(52) = .886 *p* = .380). Mean error rates were 17.86% +/− 1.57 for the GABA group and 19.89% +/− 1.66 for the PLAC group (see Fig. 2 a). Thus, the learning performance of the groups did not differ prior to treatment.

#### Performance during extinction learning and recall

For an overview of the groups’ extinction learning and recall performance after drug/placebo administration, we conducted an ANOVA with repeated measures containing the between-subjects factor “treatment” (Lorazepam, Placebo) and the within-subject factor “learning phase” (errors in extinction learning; errors (i.e. acquisition responses) in the recall phase). The ANOVA yielded a significant main effect of learning phase (F(1) = 5.501 *p* = .023; mean percent extinction errors: GABA 23.01% +/− 2.48, PLAC 16.49 +/−1.53; mean percent recall errors: GABA 23.52% +/− 3.88, PLAC 6.11% +/− 2.51) and of treatment (F(1) = 14.772 *p* = .000) as well as a significant learning phase*treatment interaction (F(1) = 6.685 *p* = .013). The data indicate that overall performance was affected by treatment with the GABA A agonist, and that performance changed over the learning phases. In addition, the interaction suggests a differential effect of the treatment upon the successive learning phases. In the following analyses, we analyzed the data in more detail, directly comparing the groups’ performance in the ABA and AAA conditions.

##### Extinction learning

The GABA group made significantly more errors than the PLAC group in extinction trials proper (i.e. in those trials in which the previously learned consequence of stomach trouble changed): *t*(52) = 2.233 *p* = .030 (mean percent errors: GABA 23.01% +/− 2.48; PLAC 16.49% +/−1.53. This higher error rate was observed in both conditions: ABA trials with consequence change (ABA CC) *t*(52) = 3.4118 *p* = .003 (mean percent errors: GABA 25.93% +/−2.64; PLAC 15.85% +/−1.85); AAA trials with consequence change (AAA CC): *t*(52) = 2.447 *p* = .018 (mean percent errors: GABA 24.77% +/− 2.69; PLAC 17.13% +/− 1.58). However, GABA participants also made more errors during trials without consequence change (distractor trials), which required retrieval of associations acquired previously: *t*(52) = 2.117 *p* = .039 (mean percent errors: GABA 20.10% +/− 3.71; PLAC 11.69% +/− 1.43). (see Fig. 2 b).

To investigate the extinction learning progress of the groups, we used an ANOVA with the repeated measures factor extinction learning block (8 blocks a 10 trials each). The analysis showed a significant main effect of learning block (F(7) = 84.025 *p* = .000 as well as a significant main effect of treatment F(1) = 9.803 *p* = .003, while the interaction was not significant. In a comparison of three phases of extinction learning (initial exposition to the changed associations - block 1; early extinction learning - block 2 to 5; and late extinction learning - block 6 to 8) we observed significant differences between the groups in their learning progress during early and late extinction learning ( early: *t*(52) = 2.305 *p* = .025, mean percent errors: GABA 21.58% +/− 2.02, PLAC 15.27% +/− 1.84; late: *t*(52) = 2.523 *p* = .015, mean percent errors: GABA 11.88% +/− 2.33, PLAC 5.35% +/− 1.26), but not during initial exposition to the changed associations (*t*(52) = 1.839 *p* = .072, mean percent errors: GABA 50.33% +/− 2.95, PLAC 42.59% +/− 2.99). Thus, both groups achieved the learning criterion of >80% correct responses during late extinction learning. Nevertheless the higher error rate of the GABA group indicates a persisting learning impairment.

##### Recall

During overall extinction recall, the GABA group responded more frequently than PLAC with associations that were correct during acquisition (*t*(52) = 3.920 *p* = .000 (percent acquisition responses GABA 23.52% +/− 3.88, PLAC 6.11% +/− 2.16). This was true also for separate analyses of the ABA and AAA conditions: we observed significant differences between GABA and PLAC participants regarding the percentage of their ABA renewal responses, *t*(52) = 3.182 *p* = .002 (percent renewal responses: GABA 31,11% +/−4.98 s.e.m.; PLAC 11.11% +/− 3.83 s.e.m.) and the percentage of their errors in AAA recall (i.e. in the condition controlling for impaired extinction retrieval), *t*(52) = 4.192 *p* = .000 (percent recall errors: GABA 15.92% +/− 3.44; PLAC 1.11% +/− 0.77). The results point towards a context-independent impairment in retrieving extinction associations in the GABA group.

In contrast, the groups did not differ regarding their error rates in retrieval of distractor trial associations, which did not change during extinction learning *t*(52) = 1.059 *p* = .295 (GABA 12.11% +/−3.76; PLAC 7.22% +/− 2.72), despite the significant between-group difference in memory for these associations during extinction learning. It is therefore conceivable that the GABA group benefitted from the additional training with these stimuli during the extinction learning phase.

#### Correlations of extinction learning and recall performance

In a further approach to determine the relation between the phases of extinction learning and recall in the two conditions, we performed correlational analyses of the error rates in extinction learning and the error/renewal rates in recall, assuming a positive correlation. In both conditions, the correlation between these parameters was significant in the GABA group (AAA: *r* = .685 *p* = .000; ABA: *r* = .388 *p* = .023), but not in the PLAC group (AAA; *r* = .275 *p* = .0825; ABA: *r* = .268 *p* = .0885), i.e. the error levels in the two phases are unrelated in untreated participants, but related in participants having received the GABA A agonist. Together, the findings on the performance of the GABA group suggest that during recall, their observed response pattern could be at least partially caused by impaired retrieval of extinction memory instead of context consideration. To further explore this possibility, we performed an additional analysis below.

#### Frequency of renewal

In the GABA group, 81.48% of all participants (*n* = 22) showed an ABA renewal effect, while 18.52% (*n* = 5) did not (χ^2^ = 10.704 *p* = .001). In the PLAC group, the number of participants showing (*n* = 11; 40.74%) or not showing renewal (*n* = 16; 59.26%) did not differ significantly (χ^2^ = .926 *p* = .336).

Of this subset, only 31.81% (*n* = 7) of participants in the GABA group showed acquisition responses exclusively in the ABA condition, while 68.18% (*n* = 15) also recovered acquisition associations during AAA recall. In contrast, in the PLAC group, 90.90% of all participants with renewal recovered acquisition associations exclusively in ABA (10 of 11), and only 1 participant (9.10%) did so also in AAA recall. Thus, the number of participants showing exclusively ABA renewal did not differ between the groups (χ^2^ = .529 *p* = .467), while the GABA group contained significantly more participants with AAA errors (χ^2^ = 13.235 *p* = .00).

Consequently, the strength of the renewal effect in ABA recall trials did not differ between the REN participants in the two groups: t(31) = 1.224 *p* = .230 (mean percentage of renewal 37.95% +/− 5.06 in the GABA group and 27.27% +/− 7.05 in the PLAC group). However, corresponding to the behavior of the complete GABA group, those GABA participants who showed renewal exhibited a stronger tendency also during AAA recall to retrieve associations that were correct during acquisition t(31) = 2.801 *p* = .009 (mean percent errors 17.95% +/− 3.94 GABA, 1.81% +/− 1.81 PLAC). (see Fig. 2 d). In summary, the behavior pattern of the GABA group suggests an impairment in retrieving the associations learned during the extinction phase, rather than genuine, context-based renewal.

#### Renewal ratio

The GABA participants’ tendency to respond with associations correct during acquisition also in AAA recall suggested that their overall recall behavior did not always reflect context consideration. Therefore, we sought to determine the degree to which their responses during extinction recall represented “genuine” renewal, i.e. the degree to which they were based on context consideration and did not result from disturbed retrieval of extinction associations. For this purpose, we compared the frequency of responses reflecting associations that were correct during acquisition in the conditions of ABA recall (experimental condition) and AAA recall (control condition to identify impaired extinction memory retrieval). For each GABA and PLAC participant who showed renewal, we calculated the ratio between such acquisition responses in ABA and AAA recall, according to the formula ABA - AAA / ABA + AAA. In this calculation, a ratio value of 1 indicates that every response of this type occurred during ABA recall and none during AAA recall - which suggests context consideration and thus genuine renewal. In contrast, a ratio value of −1 indicates that every response of this type occurred during AAA recall and none during ABA recall, suggesting rather weak memory for AAA extinction associations, with preserved memory for ABA extinction associations, but without context consideration. Higher ratios thus signal a higher probability of genuine renewal.

The mean renewal ratio for GABA participants was .521 +/− .085, while for PLAC participants it was .961 +/− .038. The values suggest that those responses that reflected associations learned during acquisition demonstrated a response strategy strongly based on context in the PLAC group, whereas in the GABA group they did not clearly reflect context consideration. The difference between the groups’ ratios was significant (t(31) = −3.543 *p* = .001). The result indicates that genuine renewal (resulting from context consideration) is reduced in the GABA group compared to PLAC, and in consequence supports the notion that the recall behavior in the GABA group is predominantly based on impaired retrieval of extinction associations. In contrast, the level of renewal observed in the PLAC group, despite being low overall, reflected genuine renewal (see Fig. 2 d).

#### Response times

During acquisition, which was performed prior to drug administration, response times did not differ between GABA and PLAC groups (*t*(52) = .991 *p* = .326, mean GABA 829 ms +/− 54, mean PLAC 766 ms +/− 36. During extinction learning and extinction recall, which were performed after drug administration, the GABA group showed significantly slower responses (extinction learning: *t*(52) = 2.803 *p* = .007, mean GABA 996 ms +/− 85, mean PLAC 718 ms +/− 51; extinction recall *t*(52) = 4.888 *p* = .000, mean GABA 885 ms +/− 62, mean PLAC 534 ms +/− 36).

However, within the GABA group we observed a significant correlation between the participants’ response times in acquisition and extinction (*r* = .383 *p* = .049), indicating that individual participants’ response times during acquisition (prior to drug administration) tended to be similar to those during extinction. This correlation was even stronger for response times in extinction and recall phase (both after drug administration) (*r* = .697 *p* = .000). The same pattern was found for the PLAC group: a significant correlation between response times in acquisition and extinction (*r* = .458 *p* = .016) and in extinction and recall (*r* = .721 *p* = .000), indicating that, overall, participants’ individual tendencies towards slower or faster responding determinated response times to a higher degree than pharmacological manipulations did.

#### Correlations between response times and performance

Across groups, response times during extinction recall showed a positive correlation with the percentage of responses representing associations learned during acquisition, both during ABA recall (*r* = .443 *p* = .001) and AAA recall (*r* = .311 *p* = .022). Also across groups, response times during extinction learning showed a positive correlation with errors in extinction (*r* = .510 *p* = .000). Interestingly, this relation is due to the PLAC group’s behavior pattern: within each group, only PLAC showed a significant correlation between response time and error rates during extinction learning *r* = .460 *p* = .016) as well as percent renewal responses during ABA recall (*r* = .470 *p* = .013). In the GABA group, none of these correlations reached significance (response times and extinction errors *r* = .325 *p* = .098; response times and renewal rate *r* = .209 *p* = .296). The data suggest that only in PLAC high error and renewal rates were associated with slower responding.

### Imaging results

#### Extinction learning

**Fig. 3:**
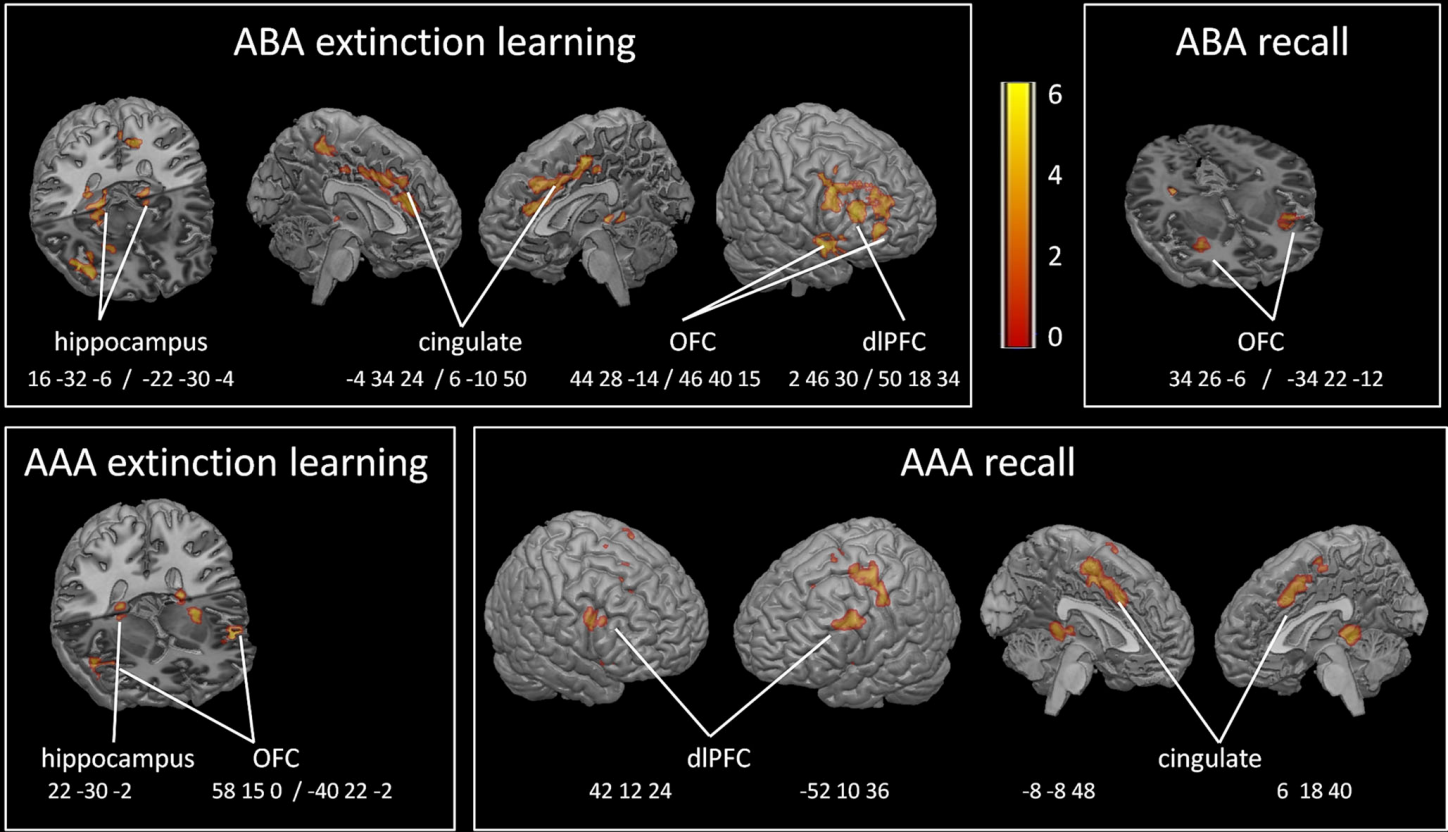
Areas of reduced activation in GABA participants compared to PLAC during extinction learning and recall in a novel (ABA) (*top row*) or familiar (AAA) context (*bottom row*). GABA agonism significantly reduced hippocampal activation during extinction learning in both conditions, compared to PLAC. Futhermore, prefrontal (OFC, dlPFC, cingulate) activation was significantly reduced in GABA participants during extinction learning and recall in both conditions. (Two-sample *t*-tests, contrast PLAC > GABA, FWE-corrected *p* < 0.05 on cluster level, minimum contiguous voxels: 10)

During ABA CC extinction learning, we observed reduced activation in the GABA group compared to PLAC in bilateral posterior hippocampus, right OFC BA 47, right superior temporal gyrus and temporal pole BA 38/22, Insula, bilateral lingual / parahippocampal gyrus BA 27/30, right lentiform nucleus, putamen, right dlPFC BA9,46 and lateral OFC BA10, moreover in left cingulate (anterior and mid) BA 24 and 32. There were no regions with increased activation in the GABA group relative to PLAC. During AAA CC extinction learning, we observed reduced activation in the GABA group compared to PLAC in: bilateral superior temporal gyrus and temporal pole BA 38, 22, right Insula, left lentiform nucleus, and putamen. Again, in the GABA group there were no regions with increased activation relative to PLAC. (Two-sample *t*-tests, contrasts PLAC > GABA and GABA > PLAC, FWE-corrected *p* < 0.05 cluster level, minimum contiguous voxels: 10). (see Table 1 and Fig. 3).

**Table 1:** Reduced activation in GABA participants during extinction learning in trials with consequence change (CC) (PLAC > GABA two-sample *t*-tests *p* < .05 FWE-corrected cluster level, minimum contiguous voxels: 10)

Region	BA	Hem	EXTINCTION ABA CC			EXTINCTION AAA CC		
			MNI coord	t-value	Voxel	MNI coord	t-value	Voxel
Dorsolateral PFC	9	R	50 18 34	6.21	82			
	46	R	32 46 30	4.55	48			
Orbitofrontal Cortex	47	R	44 28–14	6.56	48	58 15 0	3.88	13
		L				-40 22–2	3.45	30
	10	R	46 40 15	5.55	70			
Mid Cingulate	24	R	6–10 50	5.08	168			
		L	-5 -42 54	4.19	70			
Anterior Cingulate	32	L	-4 34 24	5.18	110			
Insula		R	40 10–12	4.10	29	44 14–5	3.54	20
Hippocampus		R	16–32 -6	5.50	42	22–30 -2	5.00	10
		L	-22 -30 -4	5.18	50			
Lingual gyrus		R	12–44 -4	4.56	102			
Parahippocampal gyrus	35	R	16–24 -16	3.69	92			
Superior temporal gyrus	22	R	58 12–5	5.62	137			
		L				-52 8–8	4.17	82
	43	L	-54 -12 10	4.71	31			
Temporal pole	38	R	52 16–14	6.14	107	52 16–14	4.50	33
		L				-48 18–12	4.62	57

#### Extinction recall

During ABA CC recall, we recorded reduced activation in the GABA group compared to PLAC in: bilateral orbitofrontal cortex BA 47, insula; and in right lingual, fusiform and parahippocampal gyrus. Increased activation in the GABA group compared to PLAC was seen in lefthemispheric ventromedial PFC (BA 10; peak MNI coordinate −6 58 24; cluster size 89 voxel). During AAA CC recall, reduced activation in the GABA group compared to PLAC was observed in: left hippocampus, left insula and OFC, bilateral dlPFC BA9, and left mid cingulum, right parahippocampal gyrus, bilateral lingual and fusiform gyrus. Increased activation in the GABA group compared to PLAC occurred in: bilateral ventromedial PFC (BA 10, peak MNI coordinates −4 64 16, 2 58 16; cluster size 152 voxel). (Two-sample *t*-tests, contrasts PLAC > GABA and GABA > PLAC, FWE-corrected *p* < 0.05 cluster level, minimum contiguous voxels: 10). (see Tables 2 and 3 and Fig. 3).

**Table 2:** Reduced activation in GABA participants during extinction recall for trials with consequence change (CC) (PLAC > GABA two-sample *t*-tests *p* < .05 FWE-corrected cluster level, minimum contiguous voxels: 10)

Region	BA	Hem	RECALL ABA CC			RECALL AAA CC		
			MNI coord	t-value	Voxel	MNI coord	t-value	Voxel
Dorsolateral PFC	9	R				42 12 24	3.89	10
		L				-52 10 36	4.67	78
Orbitofrontal Cortex	47	R	34 26–6	4.62	72			
		L	-34 22–12	5.06	51	-32 30–2	3.54	12
Mid Cingulate	32	L				-2 -10 46	4.50	124
	24	L				-8 -8 48	4.59	33
		R				6 18 40	4.19	49
Insula	13	L	-30 20 0	3.69	129	-32 16 2	4.98	83
Hippocampus		L				-20 -30 -4	4.30	38
Lingual gyrus	19	R	26–52–10	4.97	26	14–44 -6	4.38	92
						-18 -46 -6	4.60	71
Fusiform gyrus		R	28–52 -14	4.64	58	28–52 -14	6.53	80
		L				-28 -50 -12	4.52	57
Parahippocampal gyrus		R	16–40 -8	3.91	34	20–35 -12	4.57	43

**Table 3:** Increased activation in GABA participants during extinction recall (GABA > PLAC two-sample *t*-tests *p* < .05 FWE-corrected cluster level, minimum contiguous voxels: 10)

Region	BA	Hem	RECALL ABA CC			RECALL AAA CC		
			MNI coord	t-value	Voxel	MNI coord	t-value	Voxel
Orbitofrontal Cortex	10	L	-6 58 24	4.08	89			
Ventromedial PFC	10	R				-4 64 16	4.79	50
		L				2 58 16	4.73	100

#### Correlations of extinction performance and brain activation

Across the complete group of participants, we observed significant negative correlations between extinction performance and BOLD activation in several extinction-related brain regions during ABA extinction: high activation in left anterior cingulate (−3 34 24), right hippocampus (16–32 -6) and right dorsolateral PFC (50 18 34) was associated with low error rates during extinction learning (Spearman-Rho: left ACC −.378 *p* = .006 , right hippocampus −.360 *p* = .009, right dlPFC −.363 *p* = .008) as well as low renewal responding during recall (Spearman-Rho: left ACC −.300 *p* = .031 , right hippocampus −.336 *p* = .015, right dlPFC −.315 *p* = .023). Within the groups, there were no significant correlations between performance and activation.

## Discussion

In this study we investigated the effects of GABA A agonism upon context-related extinction learning without a fear component and upon renewal during recall of extinction memory. According to our hypothesis, the GABA A agonist lorazepam impaired extinction learning, i.e. encoding of extinction associations, both in novel and identical contexts. In addition, corresponding to our second hypothesis, renewal was reduced during recall. However, this reduction was due to a tendency to retrieve initially learned acquisition assocations in the ABA and AAA condition, instead of extinction associations, as hypothesized. Taken together, the GABA group exhibited a reduced capacity for encoding changed associations during extinction learning, thus forming weaker extinction associations, which during recall led to impaired retrieval of these extinction associations in favor of the acquisition associations learned before, a behavior reflecting reduced genuine renewal. These behavioral findings were associated with a brain activation pattern characterized by reduced activation in prefrontal and hippocampal regions, compared to placebo.

### GABA A agonism impairs extinction learning and decreases activation in prefrontal cortex and hippocampus

As evidenced by their significantly higher error rate during ABA and AAA extinction learning, the GABA group, compared to PLAC, was impaired in adjusting the associations learned during acquisition, regardless of context. In parallel, they made more errors in distractor trials, where associations did not change during extinction, which indicated collateral problems in retrieving recently acquired associations. Interestingly, even though we observed an overall deceleration of responding in GABA participants during extinction learning and recall, which presumably is an effect of the drug, their higher error rates in extinction (or renewal rates in recall) were not significantly correlated to their response times. Thus their performance impairment appeared to be a direct effect of the GABA A agonist upon brain regions processing task-relevant information, rather than a secondary effect of an overall drowsiness induced by lorazepam.

Importantly, the present behavioral findings extend the existing knowledge about the role of the GABAergic system for extinction learning, which derives from research on fear extinction in rodents (Makkar et al. 2010). Corresponding to several studies that reported impairment of extinction learning after systemic GABA A agonism (Bouton et al. 1990; DiSorbo et al. 2009; Goldman 1977; Gorman et al. 1979; Kamano 1972), we can show for the first time that the deficit is not restricted to fear extinction in animals, but also applies to human extinction learning in an associative learning task without a fear component.

Previous animal studies targeting particular brain regions showed that local hippocampal GABA A agonism in rats disrupted contextual fear extinction learning (Corcoran et al. 2005; Corcoran and Maren 2001), a result that closely corresponds to our finding of reduced hippocampal activation associated with impaired extinction learning in the GABA group. On the other hand, local prefrontal GABA A agonism in rats did not affect, even enhanced, fear extinction learning (Akirav et al. 2006; Laurent and Westbrook 2008). In contrast, the GABA A agonist-induced behavioral impairment in humans in the present study was also associated with reduced activation in dorsolateral prefrontal (ABA extinction learning) and orbitofrontal (ABA and AAA extinction learning) regions. Conceivably, low dlPFC activity in our task contributed to higher distractability, as was recently demonstrated by GABA A agonism in monkey prefrontal cortex (Suzuki and Gottlieb 2013), and impaired the ability to encode relational information between items, which has been proposed as a specific function of dlPFC (Blumenfeld et al. 2011). In addition, lateral OFC deactivation during extinction may have impaired evaluating of response options and response inhibition (Bari and Robbins 2013; Rushworth et al. 2011). In summary, these activation changes presumably caused slowed-down and less effective extinction learning in the GABA group, which is reflected in their higher error rate.

The present results complement findings from previous studies in which we tested the effects of a DA antagonist and a NA agonist upon context-related extinction learning using the same predictive learning task. Like the GABA A agonist, the DA antagonist tiapride impaired ABA extinction learning, in parallel to reduced activation in dlPFC and right hippocampus (Lissek et al. 2015b). In contrast, NA agonism enhanced extinction learning in ABA and AAA trials and increased activation in right dlPFC, vmPFC and hippocampus as well as in bilateral anterior cingulate and insula (Lissek et al. 2015b). Thus, diverse neurotransmitter systems that participate in extinction learning modulate extinction-related activation in hippocampus and prefrontal cortex - a finding which underlines the particularly prominent role of these regions in a behavioral adaptation process that requires inhibition of an obsolete response, encoding of a novel context and associating a cue with a different outcome.

### GABA A agonism reduces renewal and alters activation in several prefrontal regions during extinction recall

Since the GABA A agonist was administered prior to, and exerted considerable effects upon, extinction learning, regardless of context, it can be argued that the GABA group’s subsequent performance during recall could be - in part or completely - a result of their learning impairment during the extinction phase, and thus cannot be evaluated independently. The most peculiar aspect of the GABA group’s recall performance, compared to PLAC, and furthermore compared to results of recent studies from our lab which used the same task paradigm (Lissek et al. 2013, 2015a, b), is their high level of retrieved acquisition responses during AAA recall - a behavior that is most likely a result of impaired encoding of extinction associations. In our paradigm, the AAA condition is used to control for extinction deficits - here a high error rate during recall indicates impaired extinction learning/retrieval and thus enables relativization of performance during ABA recall. In order to specify the contributions of overall impaired extinction learning upon recall behavior, we performed an ANOVA with the repeated measures factor learning phase (extinction, recall). This analysis showed not merely an overall main effect of treatment upon extinction learning and recall, but also a significant interaction between treatment and learning phase, which indicated that the GABA agonist treatment exerted additional effects upon performance in recall, which were absent in the PLAC group.

Actually, during extinction recall, the GABA group showed a significantly higher renewal level in ABA recall than PLAC, which at first glance suggested increased context-dependent responding. However, as mentioned above, the GABA group’s error levels in the control condition of AAA recall were significantly higher too, pointing towards a strong overall tendency in GABA participants to respond with cue-outcome associations that had been correct during acquisition, regardless of context.

Provided extinction learning is successful, participants have two response options during recall in the predictive learning task: a) context-dependent responding, which yields a response pattern that shows genuine renewal by favoring associations learned during acquisition for the ABA trials and associations learned during extinction for the AAA trials, or b) context-independent responding, which results in a response pattern that regardless of context favors the associations acquired most recently, i.e. during extinction, and shows no renewal at all. In case of impaired extinction memory encoding or retrieval, a third option c) would favor responses that were correct during acquisition in both the experimental ABA and control AAA conditions.

Based on findings from animal studies, we originally hypothesized that GABA A agonism and its effects upon hippocampal processing would disturb context encoding in extinction learning and thus promote the retrieval of extinction associations in ABA recall, due to a lack of context discrimination. The observed recall behavior of the GABA group, however, showed associations learned during acquisition indiscriminately in both contexts, a pattern that points towards impaired extinction memory retrieval, as described above. Our calculation of the ratio between such responses in ABA and AAA recall indicates that renewal, i.e. responding based on context consideration, was actually reduced in the GABA group relative to PLAC. The combined data from ABA and AAA trials suggest that treatment with the GABA A agonist reduced retrieval of the most recently acquired associations in favor of retrieval of associations learned during acquisition.

This assumption is corroborated by the finding that the GABA group showed no deficits, relative to PLAC, in retrieving the correct cue-outcome associations for distractor trials, which were established during acquisition and never changed during extinction learning. Thus, across all recall trial types the GABA group frequently responded with associations firstly acquired during initial learning. This pattern of behavior may result from mere impairments in inhibition of acquisition associations, or from deficits in encoding of extinction associations, or from a combination of both, which may result in an inability of extinction associations to inhibit acquisition associations during recall. Even though encoding of extinction associations proceeded at a slower pace in GABA participants, in the final three blocks (i.e. 24 trials) of extinction their level of correct responses was around 88%, compared to around 95% in the PLAC group, which indicates that also the GABA group was eventually successful in encoding the extinction associations. However, this does not preclude the possibility that associations encoded during extinction had less strength. Thus, conceivably, the GABA group’s recall behavior was predominantly due to impaired inhibition of the acquisition associations by the more recently learned, but weaker extinction associations.

Furthermore, the GABA group’s reduced context consideration, revealed by means of the ABA/AAA recall ratio, corresponds closely to findings from a study with GABA A agonism in rat dorsal hippocampus prior to fear extinction learning: local infusion of muscimol caused context-independent responding, i.e. equal renewal of fear regardless of whether the rats were tested in the context of extinction or in a different one (Corcoran and Maren 2001). This context-independent responding evoked by inactivation of hippocampus suggests that also in our case, a lack of hippocampal input may have contributed to the observed behavioral pattern. Actually, reduced hippocampal activation was registered in GABA participants relative to PLAC throughout ABA and AAA extinction learning, suggesting disrupted context encoding, which in turn caused a lack of available context information during recall. The importance of hippocampal activation during extinction learning for context-dependent responding in recall was demonstrated in a previous study using the predictive learning task with untreated participants (Lissek et al. 2013). Participants who later showed renewal exhibited more prominent hippocampal involvement in extinction learning, indicating encoding of relevant context that later allowed for context-related retrieval of one of the two competing cue-outcome associations previously established during acquisition and extinction, respectively. However, if context is not encoded in hippocampus during learning, during recall the necessary, prefrontally-based response selection process is not supported by hippocampal context information. Thus, in many recall trials of the present study, the GABA group tended to retrieve the better encoded cue-outcome associations, regardless of context, which probably were those learned before administration of the drug. Overall, our findings suggest that for GABA participants, during recall the context delivered insufficient support for proper association retrieval/selection.

In contrast to the widespread deactivations in the GABA group compared to PLAC during ABA and AAA extinction learning and recall, circumscribed regions in orbitofrontal ventral / ventromedial PFC (BA 10) showed higher activation in ABA and AAA recall, respectively. In previous studies, activity in these regions was found relating to recall in general (Lissek et al. 2015a) and to a high level of renewal in particular (Lissek et al. 2013), suggesting that they are involved in selecting appropriate responses using hippocampal input (Young and Shapiro 2011) of contextual information. Conceivably, in the present study, this process was disrupted in GABA participants due to deficient hippocampal input, so orbitofrontal/ventromedial PFC activation in this region did not result in unambiguously context-based response selection.

In summary, the extinction recall deficit observed in the GABA group was presumably based on a two-fold effect of the GABA A agonist: on the one hand, due to a hippocampus-based lack of context encoding during extinction learning, necessary supporting context information during recall was missing; on the other hand, due to a PFC-based impairment in processing the changed associations both during learning and recall, the associations learned during acquisition were not sufficiently inhibited by the more recently learned, but presumably weaker extinction associations.

### Modulation of retrieval suppression by the GABA A agonist

A model of inhibitory control and retrieval suppression mediated by prefrontal-hippocampal pathways (Anderson et al. 2015) might contribute to explain the interplay of effects of the GABA A agonist upon behavioral performance and activation in hippocampal and prefrontal brain regions. The authors tested retrieval suppression by comparing BOLD activation in two task conditions in which participants are prompted with the first item of a pair (of previously learned paired associates) and instructed to either think of the second item (retrieval) or not to think of the second item (retrieval suppression) (Anderson et al. 2015). In such a task, successful retrieval suppression is reflected in high dlPFC (BA 9,46) activation and reduced hippocampal activation, suggesting that dlPFC inhibits or suppresses hippocampal retrieval of the second item. This activation pattern has been demonstrated for visual stimuli such as word-word, word-face, word-place paired associates (Anderson et al. 2015). The dlPFC modulation of hippocampal activity is supposed to be accomplished via involvement of anterior cingulate (in particular BA 32), and further pathways implicating entorhinal cortex (in particular BA 28) and/or the thalamic nucleus reuniens.

Evidently, for successful extinction learning and context-related recall, retrieval suppression is required also in our task design, in which visual context-cue pairs and combined cue-outcome pairs are being presented. Outcomes that were associated with context-cue pairs during acquisition have to be suppressed/inhibited for extinction to occur, while to achieve context-related recall, the retrieval of a recently learned outcome association may have to be suppressed in favor of a previously learned association. In the GABA group, we observed decreased bilateral dlPFC activation in ABA extinction learning and AAA recall. At the same time, hippocampal activation was reduced in ABA and AAA extinction learning as well as in AAA recall. In the framework of the retrieval suppression model these deficits suggest that while dlPFC capacities to suppress retrieval were reduced, hippocampal retrieval capacities were compromised despite lacking dlPFC intervention. This configuration may have contributed to the GABA group’s higher error rate in extinction learning, whose inconsistent pattern suggested impaired retrieval of previously acquired associations on the one hand (errors in distractor trials) - conceivably related to reduced hippocampal contribution, and less efficient retrieval suppression of previously acquired associations (errors in extinction trials) on the other - conceivably related to reduced dlPFC contribution. During AAA recall, impaired retrieval suppression of the firstly acquired cue-outcome association may have contributed to the higher error rate in this condition - conceivably association with reduced dlPFC activation.

### Conclusion

To the best of our knowledge, this study is the first to investigate the effects of GABA A agonism upon brain activation patterns in non-fear-related associative extinction learning in healthy humans, demonstrating that deficits related to manipulations of the GABAergic system are not restricted to fear extinction in animals. Our findings highlight the participation of the GABAergic system in hippocampus and prefrontal regions in context-related extinction learning and recall. While decreasing activation in hippocampus, ACC and dlPFC, the GABA A agonist lorazepam impaired the encoding strength of extinction associations and context during the extinction learning phase, a deficit which led to impaired retrieval of extinction memories, associated with reduced consideration of context information during the recall phase. In contrast, retrieval of associations learned during acquisition was largely unaffected, causing reduced genuine renewal, since acquisition associations were retrieved context-independently. The deficits are potentially related to impaired retrieval suppression. Overall, our results point out that GABAergic neurotransmission is involved in the adjustment of behavior to changing consequences in a context-related extinction task.
